# The Impact of Fear, Secrecy, and Stigma on Parental Disclosure of HIV Status to Children: A Qualitative Exploration with HIV Positive Parents Attending an ART Clinic in South Africa

**DOI:** 10.5539/gjhs.v5n2p49

**Published:** 2012-11-28

**Authors:** Sphiwe Madiba

**Affiliations:** 1 Department of Environmental and Occupational Health, School of Public health, University of Limpopo, Medunsa Campus, South Africa

**Keywords:** South Africa, non-disclosure, HIV status, children, parents, antiretroviral treatment

## Abstract

South Africa is one of the sub Saharan countries where considerable progress in providing antiretroviral treatment (ART) has been made. The increased access to ART contributes to improvements in the prognosis of HIV and parents are more likely to raise their children than ever before. The study examined the social context influencing disclosure of parental HIV status to children from the perspectives of fathers and mothers accessing ART from an academic hospital in South Africa. Three focus group interviews were conducted with 26 non-disclosed biological parents of children aged between 7 and 18 years. Their ages ranged between 20-60 years and they cared for a total of 60 children. Parental decision not to disclose their HIV status to children was influenced by the fear of death and dying, the influence of television and media, stigma and discrimination. Parents delayed disclosure of their HIV status to children because children believed that AIDS kills. Parents also feared that the child may not be able to keep the parent's HIV status secret and might result in the family being subjected to stigma, discrimination, and isolation. Fear of stigma and discrimination were also responsible for the continuous efforts by parents to protect their HIV status from their children, family and neighbours. Parents also delayed disclosure to children because they lacked disclosure skills and needed support for disclosure from health care providers. Healthcare providers are in a unique position to provide such support and guidance and assist parents to disclose and children to cope with parental HIV infection.

## 1. Introduction

The world health organisation estimates that over 8 million people in low and middle-income countries were receiving antiretroviral therapy (ART) in 2011. In sub-Saharan Africa treatment coverage increased by 19% between 2010 and 2011 (WHO, UNAIDS, & UNICEF, 2011). South Africa is among the countries where considerable progress in providing ART has been made. In the past eight years, the country has established the largest public-sector ART programme in the world (UNGASS, 2010). Similar to well developed countries, the increased access to ART contribute to improvements in the prognosis of HIV. As HIV related illnesses become chronic illnesses, parents are living with their illness for many years and are more likely to raise their children (Mellins et al., 2008; Pilowsky, Sohler, & Susser, 2000). However, as parents live longer and healthier lives, other challenges emerge, and disclosure of parental HIV status to children becomes an increasingly important issue. Disclosure of parental HIV status to children is associated with better adherence to clinic appointments, lower anxiety and depression, and higher social support for parents living with HIV (Mellins et al., 2008; Mellins et al., 2002; Murphy, Stritto, & Steers, 2001). Parental disclosure to adolescent children also offers opportunities to discuss safe sexual practices (Brackis-Cott, Mellins, & Block, 2003). Furthermore, disclosure of parental HIV status to children has been shown to affect the well-being of children, parents, and family positively (Qiao, Li, & Stanton, 2011).

Studies conducted prior to the availability of ART show that parents disclosed to prepare children to face parental death (Pilowsky et al., 2000). Parents disclose their HIV status to their children when their health deteriorates or when they believe that death is imminent (Lee & Rotheram-Borus, 2002; Nam et al., 2009; Pilowsky et al., 2000; Schrimshaw & Siegel, 2002; Tompkins, 2007; Vallerand, Hough, Pittiglio, & Marvicsin, 2005). Disclosure also occurs to prepare children for future HIV related illness or the death of the parent. Parents want children to know what to do in case the parent suddenly falls ill (Cott et al., 2003; Hawk, 2007; Madiba & Matlala, 2012; Schrimshaw & Siegel, 2002). Studies show that parents disclose when they are critically ill, or their health deteriorates so that they could receive help and support from their grown-up children (Kennedy et al., 2010; Madiba & Matlala, 2012; Rwemisisi, Wolff, Coutinho, Grosskurth, & Whitworth, 2008). Parents also disclose to educate their children and protect them from HIV infection (Hawk, 2007; Madiba & Matlala, 2012; Nam et al., 2009; Schrimshaw & Siegel, 2002; Vallerand et al., 2005).

In contrast, some parents conceal their HIV diagnosis from their children because they find the process of disclosing their HIV status to children challenging (Hawk, 2007). Disclosure to children represents a serious dilemma for parents who struggle with decisions about whether and how to disclose their HIV status to children (Murphy, 2008; Rwemisisi et al., 2008; Schrimshaw & Siegel, 2002). Disclosure is often delayed because parents do not know how to tell children about their HIV status, and lack skills on how to approach the disclosure event (Corona et al., 2009; Kennedy et al., 2010; Murphy et al., 2001; Rwemisisi et al., 2008). Parents also delay disclosure because they fear that children might unintentionally tell others in school or the community resulting in stigma, discrimination, and isolation for the family (Dematteo et al., 2002; Lee & Rotheram-Borus, 2002; Murphy, Roberts, & Hoffman, 2002; Ostrom, Serovich, Lim, & Mason, 2006). Mothers, in particular, conceal their HIV status to protect their children from the knowledge that their mother has a life-threatening disease. They worry that fears of maternal death would be a burden for their children (Tompkins, 2007).

The problems parents anticipate in disclosing their HIV status translate into low rates of disclosure to children in developing and well developed countries (Moore, Kalanzi, & Amey, 2008; Qiao et al., 2011; Schrimshaw & Siegel, 2002; Shaffer, Jones, Kotchick, & Forehand, 2001). Data show that the rates of parental disclosure to children vary from region to region. Disclosure rates reported in the US ranged from 20 to 67% (Qiao et al., 2011), while disclosure rates in developing countries were at a lower level; 44% in South Africa (Palin et al., 2009), 16% in Lome Togo (Moore et al., 2008), and 50% in Uganda (Rwemisisi et al., 2008). Disclosure rates also varied from study to study, studies focusing on adolescents reported higher rates (74%) while studies that included younger children revealed lower rates 30% (Nöstlinger et al., 2004; Palin et al., 2009; Pilowsky et al., 2000). Low parental HIV disclosure rates to children is also attributed to parental desire to protect children from social consequences such as stigma and psychological distress (Moore et al., 2008). As mentioned, mothers delay disclosure to protect children from worry about their mother dying. Parents believe that disclosing HIV status to children would take away the joys of being a child (Ostrom et al., 2006; Schrimshaw & Siegel, 2002; Vallerand et al., 2005). However, data show that disclosed children were glad that they had been told about parental HIV status so that they could better prepare for the future, make decisions affecting themselves, reduce mother's stress, be closer to mothers, talk openly, and be less afraid (Murphy, Roberts, & Hoffman, 2006; Tompkins, 2007).

Non-disclosure of parental HIV status to children is an issue health care providers will deal with for years to come, given the increasing survival of adults and children on ART in South Africa (Shisana et al., 2009). Similar to other settings, a number of HIV positive children are developing to maturity within families affected by HIV (Ferris et al., 2007; Kallem, Renner, Ghebremichael, & Paintsil, 2010; Tompkins, 2007). Therefore, nondisclosure of parental HIV status to children invariable means nondisclosure of the HIV diagnosis to infected children, which may result in delay for HIV testing, treatment, and care for HIV infected children. Eight years after the initiation of ART, there is limited data examining parental disclosure to children in South Africa. Findings from a recent systemic review of literature on parental disclosure show that intervention studies related to parental HIV disclosure to children were limited worldwide. The study shows that most studies on parental disclosure of HIV status to children were conducted in well developed countries, and that the geographic distribution of existing studies does not reflect the actual HIV epidemics and the global needs of parental disclosure. Furthermore, the social and cultural contexts for HIV disclosure practices of families affected by HIV in developing countries are likely to differ (Kennedy et al., 2010; Qiao et al., 2011). The aim of the study was to examine the social context influencing disclosure of parental HIV status to children in South Africa, to inform the development of interventions to support parents to disclose their HIV status to HIV negative and positive children. The study used a qualitative approach to explore the reasons for nondisclosure of parental HIV status to children from the perspectives of fathers and mothers accessing ART from an academic hospital in South Africa.

## 2. Methods and Materials

### 2.1 Study Design

We conducted focus group (FG) interviews with participants recruited from an HIV clinic of an academic hospital in South Africa. The clinic provides ART to adults since 2004. Data were collected between November 2010 and January 2011.

### 2.2 Data Collection

FG interviews were conducted by a master of public health student trained in facilitating FGs. A research assistant also trained in qualitative methods assisted with recruitment and moderation of the FGs. Two open ended FG guides, one for disclosed and one for non-disclosed parents was used for the FG interviews. The guides were developed in English and translated into Setswana, a local language spoken by most participants in the study site. Participants were recruited during their routine visit to the clinic; recruitment was done in the mornings while they awaited their turn to be seen by the doctor, and or collect medication. After information was provided about the study, participants who were interested in being a part of the study and met the inclusion criteria of being a biological parent of a child aged between 7 and 18 years were selected for the FG interviews. Participants who were biological parents of children younger than seven years were excluded from participating. We obtained a written informed consent from individual participants prior to the start of the interview. The FG interviews were conducted in Setswana and were audio recorded with the participants’ permission. FG interviews lasted for about 60 to 90 minutes. All FG interviews were conducted on the same day of recruitment while participants waited for their medication. Participants were served refreshments at the end of the interviews. A total of six FG interviews were conducted with disclosed and non-disclosed parents of children aged between 7-18 years. Each FG interview had an average of seven participants with a total of 44 participants.

Socio-demographic information of the participants and their children was collected at the end of the FG interviews using a brief self-administered tool. The tool was translated into Setswana, and the researchers assisted participants who could not read and write to complete the questionnaire. The questionnaire collected demographic information including age, level of education, marital status, employment status, date of HIV diagnosis, and period on ART. Participants provided information on their children including number of children, the age of the children, gender, the HIV status of the children, and whether or not they had disclosed their HIV status to their children.

The Medunsa Research Ethics Committee of the University of Limpopo granted ethical approval for the study. Permission to conduct the study was obtained from the hospital management of Dr George Mukhari Academic hospital. Participation in the study was voluntary, and the researchers ensured confidentiality throughout data collection. Informed consent was obtained from participants prior to the FG interviews.

### 2.3 Data Analysis

The recorded FG interviews were transcribed verbatim in Setswana by a transcriptionist and then translated into English by the lead researcher. The transcripts were reviewed for accuracy by replaying each interview recording whilst reading and translating the transcripts by the lead researcher and the author, both are well conversant with English and Setswana. Multiple readings of one transcript were undertaken by the author and the lead researcher who independently identified important words, phrases, and concepts that related to disclosure of parental HIV status to children. After reaching consensus on the definitions of the themes and sub themes that captured the essence of the participants’ experiences, a code book was developed. The transcripts were recoded if a new code emerged or an existing code was revised. NVivo version 8 was used in the application of themes to the remaining transcripts. Themes that were consistent in terms of the process of disclosure became categories.

We used various strategies to ensure trustworthiness of the FG findings; we conducted FG interviews in the local language, recorded the FG interviews, transcribed transcripts verbatim in Setswana, held peer debriefing sessions after each FG interview to discuss emerging themes, verified raw data during translation, used a computer software for analysis, and employed researcher triangulation during data analysis (Creswell, 2007; Patton, 2002).

## 3. Findings

### 3.1 Description of the Study Sample

This paper presents data from three FG interviews conducted with 26 HIV positive parents who had not disclosed their HV status to children. The age of the parents ranged between 20-60 years. Table 1 shows the demographic profile of the participants. The 26 parents cared for 60 children aged between 7 and 18 years.

**Table 1:** Socio-demographic profile of participants

Variable name	Frequency	Percent
**Age**		
**20-30**	4	15.4
**30-40**	18	69.2
**40-50**	3	11.5
**50-60**	1	3.9
**Gender**		
**Female**	18	69.2
**Male**	8	30.8
**Marital status**		
**Single**	20	77.0
**Married**	5	19.2
**Divorced**	1	3.9
**Level of education**		
**No schooling**	1	3.9
**Primary education**	7	27.9
**Secondary education**	6	23.1
**High school**	10	38.5
**Tertiary**	2	7.8
**Employment status**		
**Unemployed**	14	53.9
**Employed**	10	38.5
**Part-time**	2	7.8
**Number of children**		
**1 child**	8	30.8
**2 children**	7	26.9
**3 children**	7	26.9
**4 children**	4	15.4

### 3.2 Themes

Six main themes emerged from the data analysis; fear of death and dying, fear of stigma and discrimination, child too young, knowing how to tell, support for disclosure and intention to disclose. Table 2 is a summary of the identified themes and subthemes.

**Table 2:** Summary of identified themes

Fear of death and dying
Influence of media
Aids kills

Fear of stigma and discrimination
Protecting HIV status
Secrecy

Child too young
Reaction to disclosure

Knowing how to tell
Support for disclosure
Intention to disclose

#### 3.2.1 Fear of Death and Dying

Parents mentioned fear of death and dying as a reason they delayed disclosure to children. Parents believed that if the child knew about the parent's HIV status, they would think that their parent was going to die.

*Children know that people infected with HIV die, you become afraid to tell because the child will say “my mother is going die” so you become afraid to tell (Mother of 2 children)*.

*My 8 year old girl is in primary school, and they were teaching them about HIV the other day and she said to me “if you can have HIV mama, I can die” This is the reason why I am afraid to tell (Mother of 4 children)*.

3.2.1.1 AIDS Kills

The context of disclosure in this study occurs within an environment where children think that AIDS kills; this was an additional reason parents delayed disclosure of their HIV status to children.

*At school they might bring something showing that AIDS kills and she will say even my mother is having AIDS, which is the reason why I am afraid to tell (Mother of 2 children)*.

*They don’t think that because I am taking treatment I can live long, to them they think that he is taking treatment, he is dying. The thing that I am taking treatment and can leave for 10 years is not there, to them “AIDS kills” (Father of 3 children)*.

Some of the children and their parents experienced the death of family members from HIV related illnesses. Consequently, parents were afraid to disclose for fear that disclosure will negatively affect the child who will think that their parent is going to die too.

*My reason for not telling him is because my sister died and he was traumatized and even failed his class. He was very close to his aunt (Mother of 2 children)*.

*My child lost her uncle from this disease; she was very close to her uncle, I am afraid to tell her about myself, I feel that she will say “I keep losing people in this house” (Mother of 3 children)*.

3.2.1.2 The Influence of Media

Most of the HIV TV campaigns and educational programs were designed to educate and make people in the country aware of HIV and AIDS, but media also creates fear for parental disclosure of HIV status to children.

*My child watches HIV related dramas on TV, many of these dramas show that a person who is HIV positive end up dying. That thing is the one that makes me to be afraid to tell my child. That is the reason why I am afraid to tell (Father of 3 children)*.

*The healthcare people and the media must try by all means not to show that a person who is HIV positive ends up dying. You must also show that when a person is taking treatment, he ends up healed. Not to always show that a person with HIV ends up dead. This is the reason why we are afraid to tell our children (Father of 3 children)*.

#### 3.2.2 Fear of Discrimination

Parents delayed disclosure because of fear of stigma and discrimination. Protecting the child and the parent from HIV related stigma and discrimination was one of the key reasons parents delayed disclosure to children.

*The reason for not telling my child is that I have one child who is 7 years old. It is good and not good to tell taking into consideration the child's age and the stigma surrounding this disease (Mother of 1 child)*.

*My reason for not telling is because a child does not have a secret, we have not accepted this disease as a community. Because my child cannot keep a secret, you are going to see people looking down at me (Father of 1 child)*.

One other way of protecting the parents and children from discrimination was secrecy. Parents delayed disclosure for fear that the child would not be able to keep the HIV status of the parent secret. Parents feared that if the child tells others about the parent's HIV status the family will be subjected to discrimination.

*Eish…, you know, this child, if you tell him, he is going to tell his friends that my father is going to die; he is having AIDS (Father of 3 children)*.

*To tell you the truth, not everyone understands the disease. It does not help to tell your child and people end up hurting your child. It will hurt you coming back from work, to find your child crying after hearing of something bad about you (Mother of 1 child)*.

3.2.2.1 Protecting HIV Status

Besides secrecy, one other strategy parents used to manage stigma and discrimination was to keep their HIV status secret. Parents used different ways to protect their HIV status and treatments from children, family members and people outside their immediate families.

*When I come back with my pills, I remove the labels from the pill containers, I know my pills, I know each one. I rub the paper label off the container, I bought pill containers in which I keep my pills, and I throw the original containers in the trash bin, containers which do not have my name (Mother of 1 child)*.

*When I come back with my pills, I take them out of their containers so that they don’t see what the pills are for (Mother of 2 children)*.

To protect their HIV status, parents who did not hide their ART medication told their children that they had other

diseases other than HIV.

*I reminded her of the time when I was admitted in hospital having liver problem and said that the treatment was for that (Mother of 1 child)*.

*I told them that I have TB. They know that I have TB and they continue to think like that, I am still not cured (Father of 3 children)*.

#### 3.2.3 Child Too Young

Age and level of maturity was one of the reasons for non-disclosure of parental HIV status to children. Parents delayed disclosure to an age when the child is perceived to be able to understand and cope with learning about the HIV status of the parent.

*I thought that the child was still young and that she will never understand this disease (Father of 1child)*.

*My experience after finding out about my HIV status…, I struggled to accept, and then I ask myself that if I struggled with acceptance, what about this child, how is she going to accept (Mother of 1 child)*.

3.2.3.1 Child Reaction to Disclosure

One of the reasons parents delayed disclosure to their children was fear of a negative reaction. They believed that when children learn about their parents’ HIV status they would be hurt and react negatively to disclosure.

*The thing that makes me to be afraid to tell is that I am afraid that maybe she would think that I am going to die in the near future; she might think that maybe my mother is left with only a few days. I think she is going to be afraid all the time (Mother of 4 children)*.

*When I think about my children, they are going to be scared; the first thing I can think of is that they are going to be scared, they will be afraid that I am going to die (Father of 3 children)*.

#### 3.2.4 Knowing How to Tell

Parents reported that the reason they delayed disclosure was because they did not know how to approach the disclosure. They were also fearful that they might be asked questions they would not be able to responding to.

*I don’t know how to approach the child because HIV scares me as an adult, for her, how is she going to take the fact that her mother is sick and is HIV positive (Mother of 4 children)*.

*I want to tell, but I don’t know how to approach the whole thing, I am not able to (Father of 3 children)*.

*I think it is still early to tell them, they are going to ask questions I won’t be able to answer (Mother of 3 children)*.

#### 3.2.5 Support for Disclosure

Parents who felt inadequate in dealing with the disclosure process would welcome the support of healthcare providers to prepare them and their children for disclosure.

*We need a procedure from health providers on how to approach them, what advice to give (Father of 1 child)*.

*I wish there can be someone who can counsel them before they can be told so that they are prepared and they are not worried by the disclosure (Father of 2 children)*.

*Before they took blood, they counsel me so that I can accept. Children need to get counselling too so that they understand that what is happening. It won’t be too painful, in that way, they will accept (Mother of 4 children)*.

#### 3.2.6 Intention to Disclose

Parents planned to disclose their HIV status to their children in the future, most parents cited age as an indication of the time they intend to disclose. One of the reasons was the right age for their children to understand the disease.

*A 10 year old is still young and can tell other children when they play, I think 15 years and above is the right time (Mother of 1 child)*.

*I want to tell my child when he completes matric, he will complete matric at 19 years, and that is the age I will be telling him, he will be matured then (Mother of 2 children)*.

Some parents planned to disclose at a time when their health had improved and they were no longer critically ill. Parents believed that in this way, children can be reassured that the parent is not going to die in the near future.

*If you are going to tell them when you are feeling down, if you are always down and feel sorry for yourself, they are going to think that their mother is sick. But if you tell them when you feel ok, they are going to accept it. There is no way that they can be stressed. If you tell them being ok they are going to take it well (Mother of 2 children)*.

*In my opinion I don’t want to tell them when I am critically ill. I want to tell them while I am still strong (Father of 1 child)*.

Parents also planned to disclose in order to educate their children about the disease so that they can take care of themselves and prevent getting infected from HIV.

*My reason for telling is to give her education about the disease. I am going to tell her the truth that I am taking HIV medication, and also give her education about the disease, how it affects a person, that it affects all people, and also how she can protect herself (Father of 3 children)*.

*If my child asks me about my HIV status, I can tell her, actually she will be giving me a platform, I will tell the truth. I will explain, she will be helping me, it will mean that she is aware and understands what is happening (Mother of 2 children)*.

Parents feel that it is best that their children should hear about their HIV status from them and believe that it might have negative impact if they hear it from other sources.

*I think they must be told by me because I think the issue is right if it comes from me (Mother of 4 children)*.

*I am going to tell her myself, but I will be happy if there were people who are counselling at the local clinics, people who can train children on how to accept (Mother of 1 child)*.

On the other hand, most fathers wanted their children to learn of their father's HIV status from their mothers.

*I think her mother is the one who is supposed to tell her because they are close (father of 1 child)*.

*It is better that the children are told by their mother, I can’t do that (Father of 3 children)*.

## 4. Discussion

Parental HIV status disclosure to children occurs in a context of fear of death and dying, the influence of television and media, stigma, and discrimination. This social context influenced disclosure of parental HIV status to children, and was often the main reason parents found disclosure to children difficult. Parents delayed disclosure of their HIV status to children because children believed that AIDS kills. Pilowsky et al. (2000) argue that the construction of HIV as meaning death and dying is not only from children’ perspectives, but that mother's concern regarding talking to children about death and dying was often the reason for nondisclosure. According to Moore et al. (2008) concerns about death comes from the knowledge that HIV infection is a death sentence in African settings where ART is not easily accessible. Similarly, thoughts of fear of death and dying characterised the discussions of disclosure of parent's HIV status to children in this study, this was despite parents being on ART during the FG interviews. Some of the children and their parents experienced the death of a family member from HIV related illnesses. Consequently, parents delayed to disclose for fear that children would be more concerned that their parents may die too.

Most of television campaigns and educational programs designed to educate and create HIV and AIDS awareness, also created fear for parental disclosure of HIV status to children. Parents perceived the television and media as negatively influencing children's perceptions of HIV positive people, and were concerned that media portrays HIV as a debilitating fatal disease. Liamputtong et al. (2009) and Lyttleton (2004) also argued that the initial media campaigns in Thailand created fear of AIDS and a continuing sense of HIV stigma amongst communities.

Fear of stigma and discrimination was one of the main reasons parents delayed HIV status disclosure to children. In this study and others, stigma associated with HIV made the decision to disclose a complex process for parents (Rosalie Corona et al., 2006; Gilbert & Walker, 2010; Nelms, 2005). Most parents were concerned about disclosing their HIV status to children, and feared that the child may not be able to keep the parent's HIV status secret. Unintended disclosure of the parent's HIV status might result in the family being subjected to stigma, discrimination, and isolation. Data from several studies show similar findings (Lee & Rotheram-Borus, 2002; Moore et al., 2008; Palin et al., 2009; Schrimshaw & Siegel, 2002). Participants in this study narrated personal experiences of stigma and discrimination following disclosure of their HIV status to some family members. Liamputtong et al. (2009) reported similar incidents; PLWHI in their study had to deal with stigma in their communities, as well as their families. Participants also reported that when neighbours or community members suspected that one is taking ART, they rummaged the communal trash bins for ART bottles to confirm that someone is indeed taking ART medication. Previous studies show that attending an HIV clinic, adhering to ART medication, and receiving a nutrition packet, draw attention to PLWHI, making their HIV positive status certainly visible, and increase HIV stigma (Gilbert & Walker, 2010; Makoae et al., 2009; Thomas, Nyamathi, & Swaminathan, 2009). Makoae et al. (2009) argue that stigma may in reality be increasing at the individual level for persons on ART who are identified because of their medications and thus become targets for stigma and discrimination. Data from this study support this view.

The data further show that because of stigma and discrimination, participants went to considerable lengths to protect their HIV status from their children, family members, and curious neighbours. Our findings are in line with previous studies (Clark, Lindner, Armistead, & Austin, 2004; Silva, Rocha, Davim, & Torres, 2008) showing that HIV positive people spend considerable energy to manipulate their environments to hide their HIV diagnosis for as long as possible. In this study, participant used different strategies to keep their diagnosis secret; they rubbed off the labels from the ART bottles to hide the name of the medication, used different nameless packages for their ART medication, and took ART medication in private. Similar to other studies, parents who did not hide their ART medication, told children that they had other diseases other than HIV (Silva et al., 2008). Most parents in this study used TB as a substitute for HIV. The need to protect the HIV status has implications for parents, and that the considerable lengths undertaken to conceal parents’ HIV status from their children may jeopardize their own health (Rosalie Corona et al., 2006).

The study further found that age and level of maturity were also key considerations for nondisclosure of parents’ HIV status to children. Parents delayed disclosure because they believed that their children were too young to understand HIV. Similar to previous studies, parents believed that at a certain age, the child would be able to understand HIV and can cope with the fact that their parent is HIV positive (Armistead, Tannenbaum, Forehand, Morse, & Morse, 2001; Hawk, 2007; Kennedy et al., 2010; Ostrom Delaney, Serovich, & Lim, 2008). The young age of the child was further associated with the fear that the child would tell others about the parents HIV status, thus subject the family to stigma, discrimination and social rejection.

Consistent with current and previous studies, parents delayed disclosure because they did not know how to tell children. They were also fearful that their children may ask questions that would be difficult and shameful for them to answer. Some of the parents were not ready to answer questions related to HIV transmission and sexuality (Kennedy et al., 2010; Murphy et al., 2001; Rwemisisi et al., 2008). Parental sexuality is considered a tabooed subject of conversation between parent and child in sub-Saharan Africa. Previous studies show that parents do not discuss the sexual transmission and prevention of HIV when they disclosed their HIV to children (Moore et al., 2008; Nam et al., 2009). However, some parents in this study planned to disclose to educate their children about HIV transmission.

We further found that fathers reported inadequacy of disclosure more often and wanted their children to learn of their father's HIV status from their mothers or grandmothers. A few fathers felt that disclosure of the parental HIV status to children should be done by health care providers. Overall, parents needed support from healthcare providers to be able to disclose their HIV status to children. They needed guidance on how to approach disclosure as well as information on HIV to enable them to respond to questions that children might ask. Our findings support previous studies suggesting that parents may benefit from support and guidance from health care professionals in planning their disclosure (Armistead & Forehand, 1995; Rosalie Corona et al., 2006; Letteney, 2006). Healthcare providers are in a unique position, to provide such support and guidance in view of the expressed need for parents to be supported.

Besides gender, no other socio-demographic characteristics of the parents influenced non-disclosure in this study. The sample consisted of participants with varied ages, level of education, marital status, and employment status. However, in a quantitative study conducted in South Africa, Palin et al. (2009) found that marital status was the only variable related to disclosure, and that no differences in disclosure were observed for other maternal variables. Data from several studies show that in some cases the decision to disclose is prompted by the parents’ ill health, and that parents who disclose soon after diagnosis do so while their health is compromised (Madiba & Matlala, 2012; Nam et al., 2009; Schrimshaw & Siegel, 2002; Tompkins, 2007; Vallerand et al., 2005). In contrast, some parents in this study planned to disclose at a time when their health had improved and they were no longer critically ill. In this way, children could be reassured that the parent is not going to die in the near future. Parents in this study and others believed that a good physical state would help to reassure children that it was possible to live a normal life for some time (Schrimshaw & Siegel, 2002). Our findings support suggestion that with increased access to ART, parents are likely to delay disclosure until their health become stable (Tompkins, 2007).

All the parents planned to disclose their HIV status to their children in the future mainly to educate them about HIV transmission. This finding is similar to one of the reasons for disclosure reported in studies in well developed countries (Brackis-Cott et al., 2003; Schrimshaw & Siegel, 2002; Vallerand et al., 2005). Most parents planned to disclose when the child was old enough to understand HIV as also indicated in other studies (Armistead et al., 1999; Vallerand et al., 2005; Wiener, Battles, & Heilman, 1998). However, the age when the child was perceived to be old enough varied, parents planned to disclose when the child reaches puberty, after completing high school, and after celebrating the 21st birthday.

A further consideration was that children should hear about their parents HIV status from the parents. They believed that disclosure might have negative impact if children learn about their parents HIV status from other sources. Similar to other studies, parents planned to disclose their HIV status to their children in a reassuring manner (Rwemisisi et al., 2008). The last consideration for future disclosure was that parents and their children should be prepared for the disclosure by health care providers. Parents’ desire for their children to be prepared goes beyond the need for support for parents who felt inadequate in dealing with the disclosure process. They desired a process where children can be counselled and provided with HIV related information to help them cope with the parent's HIV status.

There are some limitations in this study; firstly, the data presented here were collected through focus group interviews, and subject to potential selection bias. Participants who volunteered to participate in the focus group interviews might be different from those who did not volunteer. One other limitation is that the study could only report on the social context influencing parental disclosure of HIV to children at one point in time. Intervention studies on parental disclosure to children would provide insight on what influences parental disclosure and how parents disclose over time.

## 5. Conclusion

The findings show that parental decision not to disclose their HIV status to children is influenced by a variety of factors. The study found that fear of stigma and discrimination negatively influenced disclosure of parental HIV status to children to a large extent. Fear of stigma, discrimination, and gossip were responsible for the continuous effort by participants to protect their HIV status. The findings have implications for the development of health service practices that would protect HIV positive people taking ART from stigma and discrimination. In an attempt to reduce HIV related stigma, the benefits of ART should be emphasized so that communities perceive taking ART in positive rather negative way.

The study also found that despite participants being on ART, the fear of death and dying characterized their lives and influenced nondisclosure to children. The heightened fear of death and dying was attributed to the initial depiction of HIV and AIDS as debilitating, frightening and fatal disease by the media. Researchers and health care providers have a critical role to play to change the negative perceptions of HIV through the development of HIV campaigns and educational programs with positive messages depicting HIV as a chronic disease that is well controlled with ART.

Parents also delayed disclosure of their HIV status to children because they lacked disclosure skills and needed support from health care providers. Parental need for support provides an opportunity for health care providers to develop interventions to prepare parents to disclose their HIV status to children. There is a need for the development of disclosure guidelines to assist parents to disclose their HIV status to children and assist children to cope with parental HIV infection. These guidelines are particularly important because some children living with HIV infected parents, may need to be informed about their own HIV diagnosis. Heath care providers need to take these factors into consideration when they develop disclosure interventions to assist HIV positive parents to disclose.
